# Determining ‘curriculum viability’ through standards and inhibitors of curriculum quality: a scoping review

**DOI:** 10.1186/s12909-019-1759-8

**Published:** 2019-09-05

**Authors:** Rehan Ahmed Khan, Annemarie Spruijt, Usman Mahboob, Jeroen J. G. van Merrienboer

**Affiliations:** 1 Islamic International Medical College, Riphah International University, Al-Mizan IIMCT Complex, Old Supreme Court Building, 274 Peshawar Rd, Rawalpindi, Pakistan; 2 School of Health Professions Education, Maastricht University, Maastricht, The Netherlands; 3 Faculty of Veterinary Medicine, Utrecht University, Utrecht, The Netherlands; 4 Institute of Health Professions Education and Research, Khyber Medical University, Peshawar, Pakistan; 5 Centre for Medical Education, University of Dundee, Dundee, UK

**Keywords:** Curriculum viability, Quality standards, Curricular problems, Curricular diseases, Curriculum evaluation, Inhibitors

## Abstract

**Background:**

A curriculum is dynamic entity and hence, metaphorically, can be considered ‘alive’. Curricular diseases may impair its quality and hence its viability. The quality of a curriculum is typically assessed against certain quality standards only. This approach does not identify the inhibitors impeding the achievement of quality standards. The purpose of this study is to identify not only standards but also inhibitors of curriculum quality, allowing for a more comprehensive assessment of what we coin ‘curriculum viability’.

**Methods:**

We performed a scoping review of ‘curriculum viability’, after which 13 articles were found eligible through a meticulous search and selection process. We first identified 1233 studies based on matching keywords, title and abstract; 36 of which met our inclusion criteria. After application of the Qualsyst criteria, two independent reviewers performed a thematic analysis of the 13 articles that remained.

**Results:**

While all studies reported on standards of quality, only two studies described both standards and inhibitors of quality. These standards and inhibitors were related to educational content and strategy, students, faculty, assessment, educational/work environment, communication, technology and leadership.

**Conclusions:**

The framework of curriculum viability thus developed will help identify inhibitors adversely affecting the curriculum viability and remaining hidden or un-noticed when curriculum evaluation is done.

**Electronic supplementary material:**

The online version of this article (10.1186/s12909-019-1759-8) contains supplementary material, which is available to authorized users.

## Background

The curriculum has no universal definition. Curriculum theory describes the basis of its development. Its four main components are aims, contents, methods of  teaching and evaluation [1]. This theory defines the basic structure of curriculum but with more research in education, anatomy of curriculum has expanded. Learning theories or paradigms have shaped the perspectives or models of curriculum from the beginning of last century. Behaviourist learning theories are based on response to a stimulus; cognitivist paradigms explain the mind-memory phenomena whereas constructivist theory explains the buildup of knowledge on the previous knowledge [2]. Based on Skinners Behaviourist theory, Tyler in 1949 gave the prescriptive model of curricular development that comprises of educational purposes and experiences, structure and evaluation of the curriculum [3]. Hilda Taba in 1962 modified Tyler’s model by producing an ‘Interactive model, which comprised of more or less of the same components, however more emphasis was laid on learning and teaching and all components (objectives, contents, learning experiences, teaching strategies and evaluative measures) interacted with each other [4]. Walker in 1971 gave the process or descriptive model of curriculum development, which is also called naturalistic model. It was based on (i) Platform (beliefs that guide curriculum developer), (ii) Deliberation (process of making decisions) and (iii) Design (organisation and structure of the curriculum) [5]. In 1986 for undergraduate medical curricula, Harden devised ten questions, which guided the practical development of a curriculum [6]. Almost during the same time Mager stressed on the needs of defining the instructional objectives used in the curricula [7]. Gagne however had stressed in 1965 on ‘conditions of learning’ and ADDIE model in early 1980’s emphasized the instructional design based on Analysis, Design, Develop, interaction and Evaluation [8]. J G van Merrienboer gave the 4C ID model explaining the learning of a task, based on learning and training phase with reflection and feedback as the corner stones [9].

With evolution of Curriculum, it can be viewed as a sophisticated blend of educational strategies, course content, learning outcomes, educational experiences, assessment, the educational environment, timetable and programme of work [10, 11]. It becomes outdated or riddled with problems if not regularly reviewed and renewed [12]. Being a dynamic entity, the curriculum can be considered *alive* and, in its ideal state, *healthy*. In humans, the standard values for being non-diabetic are less than 125 mg/dl [13], which is one of the standards to be achieved to remain healthy. If such standard is not achieved, the person will become unhealthy (diabetic). Some factors may act as *inhibitors* to the healthy state in humans and contribute to diabetes such as: eating unhealthy food and lack of exercise. Continuing in this metaphorical vein, curricula are like humans. There are curricular inhibitors that may deter them to achieve certain expectations (standards). Relevant literature in curriculum evaluation and accreditation have more emphasis on two aspects, either on setting standards [14] and seeking evidence to confirm their fulfillments or on describing clinical pictures of some curricular diseases [15]. There is a need to explore the curricular dynamics and interplay of their elements and most importantly indicate the inhibitors that contribute to the morbidity of curricula.

The traditional approach to determine a curriculum’s health condition is to evaluate its quality. Curriculum evaluation aims to determine the curriculum’s quality by comparing it *against* different national or global accreditation quality standards [16]. These quality standards in medical education curricula, for example, serve as *expectations* and may include, but are not limited to, the World Federation of Medical Education (WFME) global standards for quality improvement; Liaison Committee for Medical Education (LCME) accreditation standards; and General Medical Council’s (GMC) ‘Tomorrow’s doctor’ standards [17–20]. In this approach, quality is synonymous with the attainment of standards [21], whether they are basic minimum standards or standards of excellence [22]. Consequently, the main emphasis is on defining quality, setting quality standards, comparing them with the outcomes, and on determining the extent to which standards have been met [23, 24].

The quality of curricula can be assessed in areas of mission and objectives, educational program, assessment, students, faculty, educational resources, program evaluation, governance and administration and continuous renewal [17]. Such quality assessment, however, does not aim to detect the inhibitors that potentially interfere with the attainment of quality standards, but only serves as a checklist of what is in order and what is not. Even if a school or agency does identify the inhibitors that impede the achievement of standards, it is not a structured process that has been described in the literature.

As such, insight into the degree to which quality standards have been met may not give a true reflection of a curriculum’s health status. The curriculum may be meeting certain quality standards, but still be fraught with problems (inhibitors) that remain unnoticed without a purposeful effort to detect them [15].

That said, we can identify two approaches in curriculum evaluation, namely: the *reviewers’* approach, which aims to provide a report on the current status of the curriculum against certain standards in a judgmental perspective, and the *interpreters’* approach, which investigates *why* standards have (or have not) been met in a more analytical stance. Reviewers need only standards and evidence from practice to decide, while interpreters need to study the underlying variables that contribute to the current state of the curriculum. Interpreters are like doctors of the curriculum; they gather information to diagnose the condition from different sources.

**Fig. 1:**
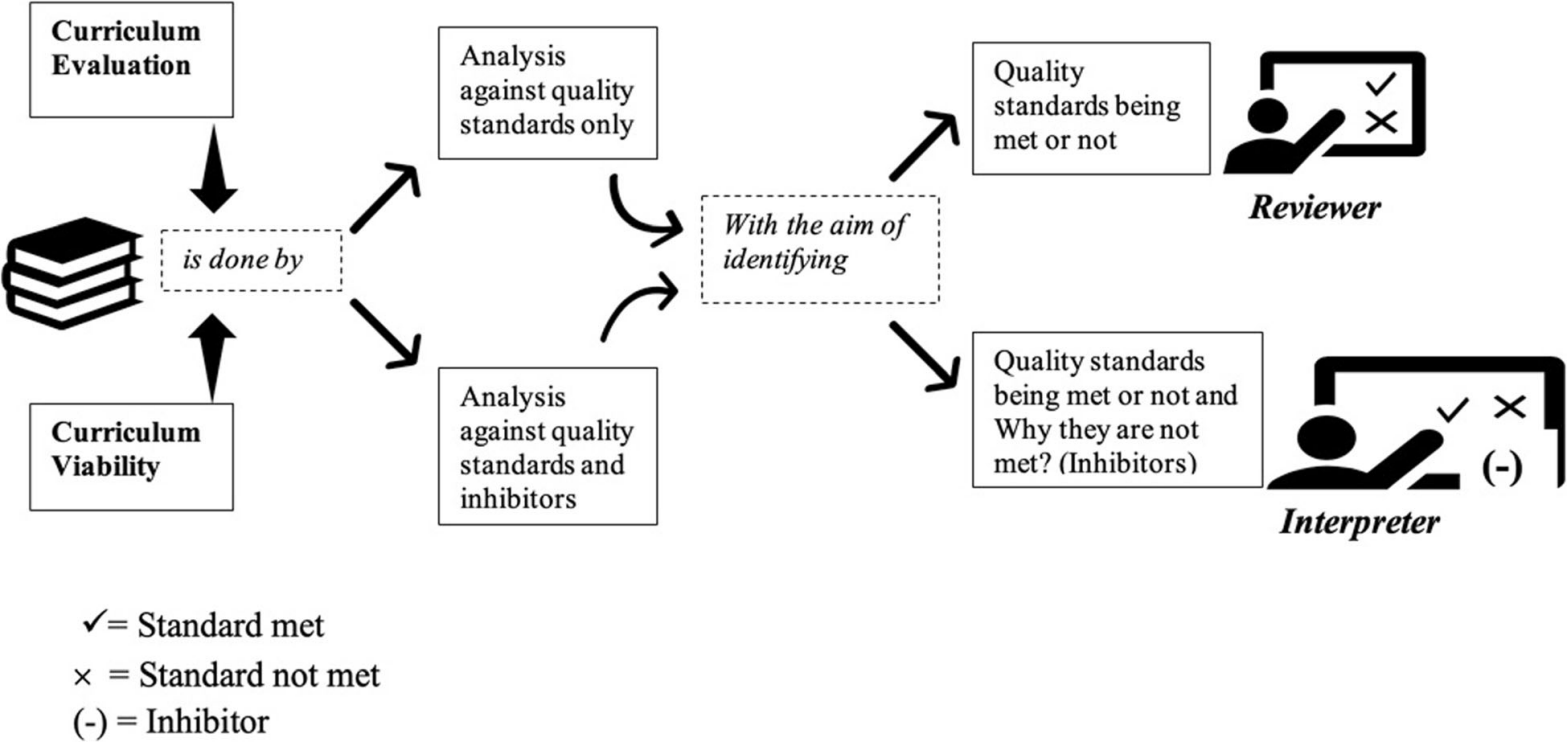
Curriculum Evaluation vs Curriculum Viability

Curriculum evaluation is done by reviewers, while we would like to introduce a new term that best suit the job of the interpreters: ‘Curriculum viability’, which is the current state of a curriculum determined by the degree to which particular quality standards have or have not been met, and inhibitors affecting the attainment of those standards. Hence, measures of viability will yield added information that is more valuable for renewal and improvement than quality measures alone. Figure 1 shows the difference in approach and outcome of curriculum evaluation and curriculum viability.

The allusion to these inhibitors is not completely new as Abrahamson in 1978 had already identified ‘Diseases of the Curriculum’ and the problems (inhibitors) responsible for them. He described nine diseases in total, along with the underlying problems in some diseases. While revisiting this iconic article, we can clearly identify some inhibitors that would help curriculum *interpreters*. For instance, c*urriculo-sclerosis* is extreme departmentalization due to extreme ownership of the subject and fighting for the hours of the discipline. *Curriculum carcinoma* is curriculum imbalance due to overgrowth of a particular curriculum segment by the disparity in the powerbase of one or more disciplines. *Curriculo-arthritis* is the miscommunication between disciplines due to limited opportunities for faculty members to meet and interact. *Iatrogenic curriculitis* is the excessive tampering with the curriculum due to abrupt and unplanned response to adjust or modify changes according to meet societal demands and expectations.

The above inhibitors clearly affect a curriculum’s viability or well-being, yet they are not considered as part of regular curriculum evaluations based on specific standards [16, 17, 25]. Sometimes the effect of inhibitors on the curriculum viability is not linear or straightforward. For instance, when faculty members resist change, this may not directly compromise curriculum quality, but it could hinder the implementation of new ideas, thereby indirectly affecting *future* curriculum reforms. Adding more sophistication, one inhibitor (e.g., ineffective communication among faculty members) may compromise different aspects of curriculum viability and contribute to different manifestations simultaneously.

In summary, we postulate that curriculum viability provides a better foundation for evaluation and improvement than do traditional quality measures and also provides a basis for preventive measures. In the current study, we planned to conduct a scoping review to provide a quick overview to identify not only standards, but also inhibitors of curriculum quality, thereby allowing for a more comprehensive assessment of *curriculum viability*. The study aims to address two research questions: [1] What, according to the literature, are standards of curriculum quality? [2] What inhibitors of curriculum quality, have been reported in the literature?

## Methods

### Search strategy

We have used scoping review as a search strategy as it is of particular use when the topic has not yet been extensively reviewed or is of a complex nature. The purpose of a scoping review is to map the body of literature on a topic area to clarifying definition and conceptual boundaries of topic or field. It shares a number of the same processes as systematic reviews as they both use rigorous and transparent methods to identify and analyze all the relevant literature [26]. Curriculum viability is a new concept, hence we selected scoping review as our methodology.

We started the scoping review by identifying and scrutinising the problem, assembling the review team and formulating research questions using Arksey O’Malley framework of scoping review [27]. Consequently, we developed a methodological and systematic search strategy by defining key terms and selecting relevant databases for our literature search. We used Web of Science (WOS) using its three data bases namely (i) Web of Science core collection, (ii) MEDLINE, (iii) SciELO citation Index. Web of Science core collection further consists of six online databases which are (i) Science Citation Index Expanded, (ii) Social Science Citation Index, (iii) Arts and Humanities Citation Index, (iv) Emerging Sources Citation Index, (v) Book Citation Index and (vi) Conference Proceedings Citation Index. So, total of eight databases were accessed through WOS. Another reason to use Web of Science was that search results are reproducible and reportable, and it contains high quality peer reviewed journals. Google Scholar was used to search for grey literature so that information that is yet to be peer reviewed is known to the researchers and also to double check that no relevant article is missed that was searched through Web of Science [28]. Other search strategies used to identify articles of interest were a manual literature search, snowballing and seeking expert help [29].

### Key terms used

To identify relevant studies, we used the key terms ‘curriculum’, ‘viability’, ‘quality’, ‘indicators’, ‘education’, ‘evaluation’, ‘issues’, ‘diseases’, ‘inhibitors’, ‘standards and tools’, in addition to the synonyms ‘syllabus’, ‘excellence’, ‘marker’, ‘teaching and learning’, ‘problems’ and ‘instruments. This yielded too many results, majority of which were not relevant to ‘curriculum viability’. The scope of search was kept broad to include the complete breadth of the topic. For this synonymous terms and Boolean Operator ‘OR’ was used. We used iterative search strategy combining different key terms to find the most relevant studies. Boolean Operator ‘AND’ was used to increase the relevance of results and to narrow them down. The details of the results of key terms and Boolean operators is given in Additional file 1.

In summary, we employed Boolean operators in concatenations of multiple keywords as in: (Curriculum OR Educational Programme OR Syllabus OR Course) AND (Indicators OR Standards) AND (Quality OR Excellence) AND (Problems OR Issues OR diseases OR Inhibitors).

### Studies selected

**Fig. 2:**
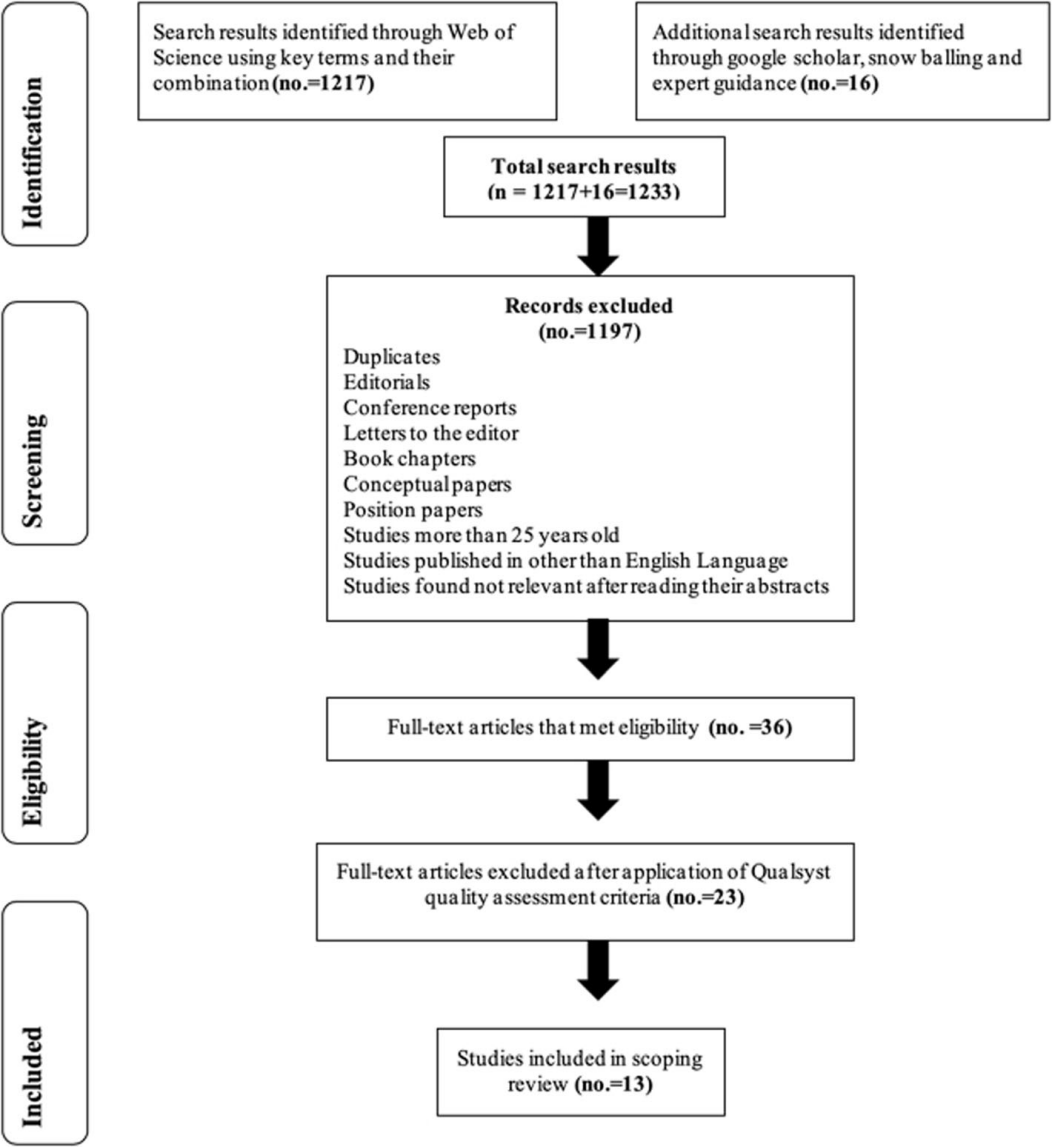
PRISMA flow diagram depicting the process of filtering articles for scoping review

The article selection process is presented in Fig. 2 and consisted of the following four phases: [1] identification, [2] screening, [3] determining eligibility, and [4] final inclusion of articles in the scoping review.

In the first phase, the lead author (RAK) identified a total of 1233 records after searching the literature for studies whose keywords, titles and abstracts matched the keywords -or combinations thereof- entered. The selection process and details were shared with the team and verified by three authors (RAK, AS & UM). We consequently applied inclusion and exclusion criteria (Table 1) and removed duplicates in the screening phase, after which 36 full-text articles remained. In the eligibility phase, these articles were read using the validated Qualsyst checklist (by RAK and UM) to assess the quality of both quantitative and qualitative studies [30], the former containing 14, and the latter comprising 10 criteria. We scored each item on a 3-point scale (0 = No, 1 = Partial, 2 = Yes) with a maximum attainable score of 28 for the quantitative and of 20 for the qualitative studies. The final score was derived by dividing the total score by either 28 or 20 or as applicable. Consistent with Kmet, Lee and Cook’s (2004) approach, we flagged a final score of > 80% as high, 71–79% as good, 50–70% as sufficient, and < 50% as limited quality. The cutoff point to include the studies in the scoping review was decided as > 50%. Thirteen articles found to be of sufficient to good quality, published in the last 25 years (from 1992 to 2017), were included in the scoping review. The inter-rater agreement using Cohen’s Kappa [31] was found to be 0.682, which is considered to be a good agreement (Additional file 2).

**Table 1:** Inclusion and exclusion criteria

Inclusion criteria	Exclusion criteria
• Original research articles and systematic reviews • Studies found through Web of Science • Studies published in last 25 years (from 1992 to 2017) • Studies related to educational research only • Studies published in English language only • Grey literature • Studies found through manual search and snowballing	• Abstracts only • Citations only • Editorials • Conference reports • Letters to the editor • Book chapters • Conceptual papers • Position papers

### Data analysis

Two authors (RAK and AS) read the 13 articles and performed a thematic analysis of the data [32]. They first used open coding to identify standards as well as inhibitors of quality, before proceeding to axial and selective coding in order to find relationships and commonalities between codes and generate themes and subthemes.

## Results

The 13 articles of sufficient to good quality finally included in the scoping review consisted of realist reviews, mixed-methods research studies, archival research, evaluation research and descriptive surveys. These articles are subsumed in Tables 2 and 3. While 11 articles specifically focused on standards of curriculum quality (Table 2), answering our first research question, only two articles covered inhibitors affecting curriculum quality (Table 3), touching upon our second research question. More specifically as is evident from Table 2, articles 1–2 discussed quality indicators for distance learning; articles 3–5 addressed quality, quality culture and quality assurance of the curriculum; article 6 concentrated on the quality of the educational environment; and articles 7–11 looked into the assessment of curriculum quality based on global WFME quality standards. Articles 1–2 in Table 3, on the other hand, specifically dealt with inhibitors of curriculum quality.

**Table 2:** Standards of curriculum quality as reported in the literature

Article	Standards of Curriculum Quality (key findings)
1. A Primer on Quality Indicators of Distance Education [33]	• Prompt feedback • Student support services • Programme evaluation and assessment • Clear analysis of audience • Documented technology plan • Course structure guidelines • Active learning techniques • Respect for diverse learning styles • Faculty support services • Strong rationale for distance education that correlates to the mission of the institution • Appropriate tools and media • Reliability of technology • Course structure guidelines • Implementation of guidelines for course development • Review of instructional materials • Institutional support and services
2. Developing of indicators of an e-learning benchmarking model for higher education institutions [34]	• Institution and organisation • Curriculum and instructional design • Resources and information technology • Learning and teaching • Learner faculties and supporting personnel • Measurement and evaluation
3. Understanding Quality Culture in Assuring Learning at Higher Education Institutions [35]	• Development of a relevant mission and vision • Achievement of internal/external standards and goals • Procurement of resources for optimal institutional functioning • Degree to which student complaints are addressed • Competence of instructors • Student engagement with faculty, staff and administration
4. Counting quality because quality counts: differing standards in master’s in medical education programme [36]	• Modality, time frame and core teaching team of the taught component • Length of programme • Length of dissertation and time allotted for its completion
5. Quality Assurance in Higher Education: A Review of Literature [37]	• Involvement of students in quality assurance process: student’s evaluation of academic programmes • Faculty-student interaction
6. Development and validation of the Dundee Ready Education Environment Measure (DREEM) [38]	• Students’ perceptions of teaching • Students’ perceptions of teachers • Students’ perceptions of atmosphere • Students’ academic self-perception • Students’ social self-perception
7. Designing an evaluation framework for WFME basic standards for medical education [14] 8. Preparing for an institutional self-review using the WFME standards – An International Medical School case study [17] 9. Evidence-based postgraduate training. A systematic review of reviews based on the WFME quality framework [39] 10. Evaluating a master of medical education programme: Attaining minimum quality standards? [40] 11. The importance of medical education accreditation standards [41]	• Mission and objectives • Educational programme • Assessment of students • Students • Academic staff/faculty • Educational resources • Programme evaluation • Governance and administration • Continuous renewal

**Table 3:** Indicators of curriculum viability, comprising standards as well as inhibitors of curriculum quality, that have been reported in the literature

Article	Curriculum viability indicators
1. Unravelling quality culture in higher education: a realist review [42]	Standards of Curriculum quality • Strategy for continuous improvement • Quality management systems • Staff and student involvement in organisational decision-making • Consideration of evolving student demands • Clear policies, procedures, systems, responsibilities • Flexible, people-oriented cultures • Presence of various cultures • Shared (educational) quality values • Leadership commitment and skills • Allocation of resources • Creation of partnerships, leaders’ ability to influence people and process management • Creation of climate of trust and sharedunderstanding • Ability to perform multiple roles • Setting and communication of policies • Communication/information for quality • Provision of information on strategies and policies • Clear task requirements and responsibilities Inhibitors of Curriculum quality • Lack of staff and student involvement in organisational decision-making • Failure to respond to evolving student demands • Lack of policies, procedures, systems, responsibilities • Lack of resources • Rigid, control-oriented cultures • Top-down approaches to quality management implementation • Presence of strong disciplinary cultures • Research culture that undervalues education • Focus on inspection and control • Leaders acting as information gatekeepers • No/insufficient sharing of best practices across the organisation • Lack of appropriate communication channels
2. Implementing an online curriculum for medical education: examining the critical factors for success [43]	Standards of Curriculum Quality • Curriculum design • Instructional feedback • Curriculum implementation • Media features • Integration • Time • Learner-centred environment Inhibitors of Curriculum quality • Inappropriate level of curriculum content • Low-quality quizzes • Technological barriers • User interface barriers • Low-quality integration • Perceived lack of sufficient time • Trainee resistance to new curriculum • Lack of social interaction

Correspondingly, two main themes emerged from the thematic analysis of these 13 articles: ‘Standards of curriculum quality’ and ‘inhibitors of curriculum quality’ addressing curriculum viability. These two themes, moreover, spanned eight subthemes or areas affecting the quality of the curriculum, specifically: 1) educational strategy and content, 2) students, 3) faculty, 4) assessment, 5) educational environment, 6) curricular communication, 7) technology, and 8) leadership. Based on these results, we created a framework to assess curriculum viability (Table 4).

**Table 4:** Framework for Assessing Curriculum Viability (Designed based on results of Tables 2 and 3)

Area	Standards	Inhibitors
Educational Strategy	1. Development of relevant Mission and Objectives 2. Curriculum design 3. Length of program 4. Implementation guidelines 5. Review of instructional material	1. Low quality integration 2. In appropriate curriculum content level
Students	6. Perception of teaching 7. Perception of teachers 8. Perception of atmosphere 9. Academic self-perception 10. Social self-perception 11. Student support services 12. Student engagement with faculty, staff and administration 13. Degree to which student complaints are addressed 14. Active learning techniques 15. Clear analysis of audience	3. Lack of time for sufficient studying 4. Neglecting Student demands 5. Student’s resistance to new curriculum
Faculty	16. Ability to perform multiple roles 17. Competence of instructors 18. Staff involvement in organizational decision making 19. Faculty Development 20. Respect Diverse ways of learning	6. Lack of staff involvement in organizational decision making
Assessment	21. Prompt feedback 22. Measurement and Evaluation	7. Low quality quizzes
Educational and working Environment	23. Flexible people-oriented culture 24. Presence of various cultures 25. Climate of trust and shared understanding 26. Learner centered environment	8. Rigid, control-oriented cultures 9. Presence of strong disciplinary cultures 10. Research culture undervaluing education
Communication	27. Communicating policies and strategies 28. Communication/Information for quality	11. Lack of sharing best practices across the organization 12. Lack of appropriate communication channels 13. Lack of social interaction
Technology	29. Documented technology plan 30. Appropriate tools and media 31. Reliability of technology 32. Resources and information of technology	14. Technology Barriers 15. User interface Barriers
Leadership	33. Create partnerships 34. Influence people management 35. Achieving internal/external standards and goals 36. Procuring resources for optimal institutional functioning 37. Allocate resources	16. Lack of policies, procedures, systems and responsibilities 17. Lack of resources 18. Acting as communication gatekeepers 19. Focus on inspection and control

### Theme 1: standards of curriculum quality

An analysis of the literature unearthed several factors that promote curriculum quality. Standards of a sound *educational strategy and content* contributing to curriculum quality were the presence of a robust and relevant mission and related objectives [35, 39, 41], a proper design, availability of instructional development and implementation guidelines, and regular reviews of instructional materials [33]. *Students* also played an important role in determining curriculum quality, with their perceptions of teaching, teachers, educational atmosphere, academic self-perception and social self-perception bearing a positive relationship to the quality of the educational environment [38]. Involving students in the organisation’s decision-making processes, evaluating academic programmes [37], letting them use active learning techniques in the case of distance learning programmes [33], and allowing them sufficient protected time [43] were all factors conducive to quality. In a similar fashion, involving *faculty* in multiple roles, organisational decision-making [42] and in regular faculty development activities, and acquainting them with new teaching methods helped boost the quality of the curriculum. Also, teachers who respected their students’ different learning approaches were important contributors to curriculum quality [17].

In the area of *student assessment,* provision of prompt feedback raised curriculum quality by affording students the opportunity to become aware of their shortcomings and improve themselves [34]. The *environment* too, could be beneficial when learner-centred as this increased learning opportunities for students [43]; and in the case of the work environment, when characterised by a flexible and people-oriented culture, a climate of trust, and a shared understanding among faculty and support staff of educational principles used in the curriculum [42]. Other quality-enhancing factors were proper *communication* of the curriculum to stakeholders which increased its effectiveness [39, 42], use of reliable *technology* underpinned by a well-documented technology plan, and selection and use of appropriate tools and media in the case of distance-based learning [33]. Finally, effective *leadership* could drive curriculum quality, with leaders having the multifaceted capacity to create partnerships, allocate resources, influence people, process management, optimise institutional functioning and achieve standards and goals [42].

### Theme 2: inhibitors of curriculum quality addressing curriculum viability

As briefly touched upon previously, only Bendermacher et al. [42] and Olson et al. [43] (Table 3) described both standards and inhibitors of curriculum quality, thereby addressing curriculum viability and answering our second research question. While Bendermacher et al. specifically focused on the organisational context elements such as communication and leadership that impact quality culture, Olson et al. explored standards and inhibitors from a distance education perspective.

Addressing the quality inhibitors, the first ones we encountered in the area of *educational strategy and content* were inappropriate content and a low level of integration preventing the proper utilisation of curriculum contents [43]. Moreover, little social interaction and *students’* resistance to curriculum renewal, acted as barriers to learning. Further undermining curriculum quality, by detrimentally affecting quality culture, was a failure to respond to evolving student demands [42]. In distance learning programmes, a perceived lack of sufficient time caused by excessive service obligations, a lack of protected time and infringes on personal time all compromised curriculum quality [43]. Other impediments to curriculum quality were the exclusion of *faculty* from organisational decision-making [42] and an unfavourable *educational/work environment*. The latter denotes a rigid, control-oriented and disciplinary culture and a research culture that undervalues education [42].

While weak *communication* channels and lack of sharing of best practices across the organisation obscured the attainment of good curriculum quality [42], *technological* hindrances, such as software problems during video-enhanced lectures and insufficient computer access at work did the same in the case of distance learning programmes [43]. To end, ineffective *leadership* could adversely affect the quality of the curriculum, which was the case when educational leaders failed to establish clear policies, procedures, systems, resources and a distribution of responsibilities, and/or acted as communication gatekeepers who focused on inspection and control only [42].

## Discussion

The purpose of the present scoping review was to identify standards and inhibitors of curriculum quality to assess curriculum viability. Since the assessment of a curriculum’s viability requires knowledge of both standards and inhibitors of curriculum quality, we made an effort to find articles that described both elements. We found 13 studies in total of which only two studies [42, 43] fully met this criterion, although they did not specifically refer to these factors as assessing curriculum viability.

In our quest for standards of curriculum quality that would answer our first research question, we found different definitions and explanations of such standards in the research literature. Different quality standards appeared to exist, but the ones most often cited in the papers we reviewed, were the widely accepted WFME global standards, which are structured according to nine broad areas of curriculum quality [22]. Although these standards enjoy wide currency and offer a holistic representation of quality, many find them difficult to interpret and use [14]. Another disadvantage is that they make no reference whatsoever to the inhibitors potentially affecting the curriculum quality. Next, a few papers included in our scoping review specifically addressed quality standards for distance learning curricula [33, 34, 43]. Comparison of the quality standards for distance-based curricula with those of their campus-based counterparts led us to conclude that the former, albeit sparse, varied and less structured, harboured the additional quality areas of ‘technology’ and ‘protected time’.

As previously mentioned, Bendermacher et al. [42] and Olson et al. [43] provided an indirect answer to our second research question. In their realist review, Bendermacher et al. described the organisational context elements that inhibit quality culture. Organisational context is one of the areas that affects the curriculum quality. Harden in 1986, in his ‘*Ten Questions’* also describes ‘Organisation of the curriculum’ as one of the questions to be answered while developing the curriculum [6]. Regarding organizational context, leadership and communication are important areas [17], without which successful implementation of curriculum is not possible as they affect the quality of curriculum directly. Related to this, Bendermacher et al. have described the inhibiting elements related to ineffective leadership, lack of student and staff involvement, insufficient resources, a rigid culture and poor communication. Olson et al. on the other hand, only addressed quality determinants pertaining to distance learning.

While it is true that reference to inhibitors related to the development, integration, content and communication of curricula is not new [15], we did not find any recent literature on viability indicators that combined standards *and* inhibitors of curriculum quality in the areas of ‘mission and objectives’, ‘faculty development’, ‘student assessment’, ‘student support’, ‘governance’, ‘programme evaluation’ and ‘curriculum renewal’. Early detection of inhibitors in these areas may help prevent a curricular catastrophe from developing. Since prevention is better than cure, any curriculum assessment should not neglect to identify the inhibitors that potentially deter the attainment of desired standards.

Based on the results previously outlined, we developed a framework for curriculum viability (Table 4) that combines 37 standards and 19 inhibitors. Taken together, standards and inhibitors can be considered as ‘indicators of curriculum viability’. They have been divided among the eight subthemes or areas affecting the quality of a curriculum, namely: educational strategy/content, students, faculty, assessment, educational/work environment, curriculum communication, technology and leadership. The areas identified are comparable to components of the curriculum as reported in literature [6, 17, 44] and closely matches the WFME standards that have wider acceptance and used in undergraduate medical curriculum [14]. This would help the evaluators to use a familiar process of evaluation.

Inhibitors against standards have been scantly reported in the literature; hence our curriculum viability framework has only considered those standards against which inhibitors were reported directly or indirectly in an impact factor journal. This is being reflected in the framework where there are greater numbers of standards in one area than in another area, and the same is true for inhibitors.

Curriculum viability framework will enable the evaluators to assess the curriculum not only for standards but for inhibitors as well. This will help them link the existing inhibitors that may be impeding the achievement of curriculum standards and also provide the ‘prophylaxis’ to avoid the development of issues in the curriculum. It will also support the process of curriculum mapping, which is an effective way to find the gaps between the developed (the designed or the official curriculum), implemented (functional or the taught curriculum) and the learned (assessed) curriculum. Mapping identifies the gaps between these phases of curriculum by establishing, relating and analyzing links between different components of the curriculum [45]. However, it does not explicitly find the reasons behind these gaps. Using the curriculum viability framework alongside the mapping process, it will help the curriculum expert to look for gaps in different areas where inhibitors have already been identified.

The framework is mainly intended towards interpreters who aim to find possible causes of an ‘unhealthy curriculum’; either to prevent it from the disease or cure it. For instance, ‘students’ resistance to new curriculum’ will not only explain the impediment of achievement of standards in the area of ‘students’, such as ‘lack of student engagement with faculty’, but it may also effect other areas such as ‘assessment’. Hence the inhibitors may not only be interpreted against their standards but also as stand-alone problems that may afflict any area of the curriculum. The timely identification of these inhibitors prevents the curriculum from becoming less viable or non-viable.

### Limitations

One of the limitations of this study is that we were unable to establish links between standards and inhibitors of curriculum quality in a holistic fashion. This is because the literature on inhibitors of curriculum quality was scant. However, our analysis of the literature did result in a framework presenting indicators of curriculum viability that embraces both standards and inhibitors. This framework as depicted in Table 4 may guide the further exploration of inhibitors in curriculum areas hitherto uninspected, that potentially explain why particular standards have not been met. The second limitation was that we searched for English-language articles only, meaning that we may have missed some studies on curriculum quality and viability written in other languages.

### Future recommendations

Curriculum issues can be diagnosed using different techniques, models and tools. These may include Harden’s Ten Questions [6], Posner Analysis [46], Kern six steps of developing curriculum [44], SPICES model [47] and bench marking the curriculum against any set criteria such as WFME [48], LCME (Liaison Committee for Medical Education [49] and Tomorrow’s doctor 2011 [50]. In this respect where an institute is aiming to diagnose curricular issues, no systematic tool or inventory to measure curricular problems has been reported in the literature. It is important that instruments be developed that measure not only a curriculum’s quality but also its viability, enabling stakeholders to obtain a true and comprehensive picture of their curriculum’s current health status and to identify the reasons why specific standards have not been met.

## Conclusion

This scoping review explored different standards and inhibitors of curriculum quality. We introduced the term ‘curriculum viability’ as denoting a curriculum’s well-being that can be determined only by considering the degree to which quality standards have been met or not attained as well as the inhibitors affecting the attainment of those standards. We hope that this modified evaluation framework will help identify problems adversely affecting the well-being of a curriculum and remaining hidden or un-noticed when curriculum evaluation is done, thereby contributing to its improvement and innovation.

## Additional files

Additional file 1: (Online Search Strategy). (DOCX 24 kb)

Additional file 2 Assessing the quality of articles and Inter-rater agreement. (DOCX 2207 kb)

## Data Availability

The data generated and analysed during the study are through research articles that have been referenced and can be accessed through the reference list.

## Abbreviations

DREEM: Dundee Ready Education Environment Measure
GMC: General Medical Council
LCME: Liaison Committee for Medical Education
WFME: World Federation of Medical Education
WOS: Web of Science
