# Supplementary material for: Determining ‘curriculum viability’ through standards and inhibitors of curriculum quality: a scoping review
Source: BMC Med Educ. 2019 Sep 5;19:336. doi: 10.1186/s12909-019-1759-8 (PMC6727426; doi:10.1186/s12909-019-1759-8)
Supplement: Supplementary file 1 — (Online Search Strategy). (DOCX 24 kb) [file 12909_2019_1759_MOESM1_ESM.docx]

**Additional file 1: (Online Search Strategy)**

Data bases: Web of Science including its three data bases namely (i) Web of Science core collection (ii) MEDLINE (iii) SciELO citation Index.

*Web of Science core collection* further consists of six online databases which are (i)Science Citation Index Expanded, (ii)Social Science Citation Index, (iii)Arts and Humanities Citation Index, (iv)Emerging Sources Citation Index, (v) Book Citation Index and (vi)Conference Proceedings Citation Index.

| S.No | Search Results | Key terms and their Combinations |
| --- | --- | --- |
| # 1 | 104,285 | (Teaching and learning) |
| # 2 | [3,550](http://apps.webofknowledge.com/summary.do?product=UA&doc=1&qid=125&SID=C4wLCyWhPfpFS8aSDPh&search_mode=GeneralSearch&update_back2search_link_param=yes) | (syllabus) |
| # 3 | 796,450 | (course) |
| # 4 | [99,876](http://apps.webofknowledge.com/summary.do?product=UA&doc=1&qid=127&SID=C4wLCyWhPfpFS8aSDPh&search_mode=GeneralSearch&update_back2search_link_param=yes) | (Educational Program) |
| # 5 | [25,709](http://apps.webofknowledge.com/summary.do?product=UA&doc=1&qid=128&SID=C4wLCyWhPfpFS8aSDPh&search_mode=GeneralSearch&update_back2search_link_param=yes) | (Excellence) |
| # 6 | [2,121,622](http://apps.webofknowledge.com/summary.do?product=UA&doc=1&qid=129&SID=C4wLCyWhPfpFS8aSDPh&search_mode=GeneralSearch&update_back2search_link_param=yes) | (Standard) |
| # 7 | [984,578](http://apps.webofknowledge.com/summary.do?product=UA&doc=1&qid=130&SID=C4wLCyWhPfpFS8aSDPh&search_mode=GeneralSearch&update_back2search_link_param=yes) | (marker) |
| # 8 | [2,344,336](http://apps.webofknowledge.com/summary.do?product=UA&doc=1&qid=131&SID=C4wLCyWhPfpFS8aSDPh&search_mode=GeneralSearch&update_back2search_link_param=yes) | (Problems) |
| # 9 | [1,295,140](http://apps.webofknowledge.com/summary.do?product=UA&doc=1&qid=132&SID=C4wLCyWhPfpFS8aSDPh&search_mode=GeneralSearch&update_back2search_link_param=yes) | (Issues) |
| # 10 | [9,747,022](http://apps.webofknowledge.com/summary.do?product=UA&doc=1&qid=133&SID=C4wLCyWhPfpFS8aSDPh&search_mode=GeneralSearch&update_back2search_link_param=yes) | (Diseases) |
| # 11 | [512,067](http://apps.webofknowledge.com/summary.do?product=UA&doc=1&qid=134&SID=C4wLCyWhPfpFS8aSDPh&search_mode=GeneralSearch&update_back2search_link_param=yes) | (Instruments) |
| # 12 | [130,817](http://apps.webofknowledge.com/summary.do?product=UA&doc=1&qid=135&SID=C4wLCyWhPfpFS8aSDPh&search_mode=GeneralSearch&update_back2search_link_param=yes) | (curriculum) |
| # 13 | [2,402,543](http://apps.webofknowledge.com/summary.do?product=UA&doc=1&qid=136&SID=C4wLCyWhPfpFS8aSDPh&search_mode=GeneralSearch&update_back2search_link_param=yes) | (Quality) |
| # 14 | [538,731](http://apps.webofknowledge.com/summary.do?product=UA&doc=1&qid=137&SID=C4wLCyWhPfpFS8aSDPh&search_mode=GeneralSearch&update_back2search_link_param=yes) | (Indicator) |
| # 15 | [2,044,220](http://apps.webofknowledge.com/summary.do?product=UA&doc=1&qid=138&SID=C4wLCyWhPfpFS8aSDPh&search_mode=GeneralSearch&update_back2search_link_param=yes) | (Evaluation) |
| # 16 | [240,771](http://apps.webofknowledge.com/summary.do?product=UA&doc=1&qid=139&SID=C4wLCyWhPfpFS8aSDPh&search_mode=GeneralSearch&update_back2search_link_param=yes) | (Viability) |
| # 17 | [1,098,382](http://apps.webofknowledge.com/summary.do?product=UA&doc=1&qid=140&SID=C4wLCyWhPfpFS8aSDPh&search_mode=GeneralSearch&update_back2search_link_param=yes) | (Education) |
| # 18 | [1,777,173](http://apps.webofknowledge.com/summary.do?product=UA&doc=1&qid=141&SID=C4wLCyWhPfpFS8aSDPh&search_mode=GeneralSearch&update_back2search_link_param=yes) | (Inhibitors) |
| # 19 | [1,255,717](http://apps.webofknowledge.com/summary.do?product=UA&doc=1&qid=142&SID=C4wLCyWhPfpFS8aSDPh&search_mode=GeneralSearch&update_back2search_link_param=yes) | (Tools) |
| # 20 | [947,050](http://apps.webofknowledge.com/summary.do?product=UA&doc=1&qid=143&SID=C4wLCyWhPfpFS8aSDPh&search_mode=CombineSearches&update_back2search_link_param=yes) | #12 OR #4 OR #3 OR #2 |
| # 21 | [37,643](http://apps.webofknowledge.com/summary.do?product=UA&doc=1&qid=144&SID=C4wLCyWhPfpFS8aSDPh&search_mode=CombineSearches&update_back2search_link_param=yes) | #20 AND #18 |
| # 22 | [118](http://apps.webofknowledge.com/summary.do?product=UA&doc=1&qid=145&SID=C4wLCyWhPfpFS8aSDPh&search_mode=CombineSearches&update_back2search_link_param=yes) | #18 AND #12 |
| # 23 | [13](http://apps.webofknowledge.com/summary.do?product=UA&doc=1&qid=146&SID=C4wLCyWhPfpFS8aSDPh&search_mode=CombineSearches&update_back2search_link_param=yes) | #18 AND #13 AND #12 |
| # 24 | [12,705,703](http://apps.webofknowledge.com/summary.do?product=UA&doc=1&qid=147&SID=C4wLCyWhPfpFS8aSDPh&search_mode=CombineSearches&update_back2search_link_param=yes) | #10 OR #9 OR #8 |
| # 25 | [35,813](http://apps.webofknowledge.com/summary.do?product=UA&doc=1&qid=148&SID=C4wLCyWhPfpFS8aSDPh&search_mode=CombineSearches&update_back2search_link_param=yes) | #24 AND #12 |
| # 26 | 0 | #18 AND #16 AND #13 AND #12 AND #10 AND #6 |
| # 27 | 0 | #18 AND #16 AND #14 AND #13 AND #12 |
| # 28 | [25](http://apps.webofknowledge.com/summary.do?product=UA&doc=1&qid=151&SID=C4wLCyWhPfpFS8aSDPh&search_mode=CombineSearches&update_back2search_link_param=yes) | #16 AND #13 AND #12 |
| # 29 | [4](http://apps.webofknowledge.com/summary.do?product=UA&doc=1&qid=152&SID=C4wLCyWhPfpFS8aSDPh&search_mode=CombineSearches&update_back2search_link_param=yes) | #14 AND #13 AND #12 AND #5 |
| # 30 | 0 | #29 AND #16 |
| # 31 | [420](http://apps.webofknowledge.com/summary.do?product=UA&doc=1&qid=154&SID=C4wLCyWhPfpFS8aSDPh&search_mode=CombineSearches&update_back2search_link_param=yes) | #14 AND #13 AND #12 |
| # 32 | [200](http://apps.webofknowledge.com/summary.do?product=UA&doc=1&qid=155&SID=C4wLCyWhPfpFS8aSDPh&search_mode=CombineSearches&update_back2search_link_param=yes) | #16 AND #12 |
| # 33 | [2,142,295](http://apps.webofknowledge.com/summary.do?product=UA&doc=1&qid=156&SID=C4wLCyWhPfpFS8aSDPh&search_mode=CombineSearches&update_back2search_link_param=yes) | #6 OR #5 |
| # 34 | [108](http://apps.webofknowledge.com/summary.do?product=UA&doc=1&qid=157&SID=C4wLCyWhPfpFS8aSDPh&search_mode=CombineSearches&update_back2search_link_param=yes) | #33 AND #13 AND #12 AND #9 AND #8 |
| # 35 | 0 | #18 AND #13 AND #12 AND #5 |
| # 36 | [850](http://apps.webofknowledge.com/summary.do?product=UA&doc=1&qid=159&SID=C4wLCyWhPfpFS8aSDPh&search_mode=CombineSearches&update_back2search_link_param=yes) | #14 AND #13 AND #4 |
| # 37 | 0 | #18 AND #13 AND #12 AND #9 AND #6 |
| # 38 | 340 | (Curriculum AND Quality AND Indicators AND Education) |
| # 39 | [236](http://apps.webofknowledge.com/summary.do?product=UA&doc=1&qid=67&SID=C4wLCyWhPfpFS8aSDPh&search_mode=GeneralSearch&update_back2search_link_param=yes) | (Curriculum AND Quality AND Indicators AND Education)  Refined by RESEARCH AREAS (EDUCATIONAL RESEARCH OR PSYCHOLOGY) |
| # 40 | [8](http://apps.webofknowledge.com/summary.do?product=UA&doc=1&qid=68&SID=C4wLCyWhPfpFS8aSDPh&search_mode=GeneralSearch&update_back2search_link_param=yes) | (Syllabus AND Quality AND Indicators AND Education |
| # 41 | [2](http://apps.webofknowledge.com/summary.do?product=UA&doc=1&qid=69&SID=C4wLCyWhPfpFS8aSDPh&search_mode=GeneralSearch&update_back2search_link_param=yes) | (Syllabus AND Quality AND Indicators AND Education)  Refined by RESEARCH AREAS (EDUCATIONAL RESEARCH OR PSYCHOLOGY) |
| # 42 | [476](http://apps.webofknowledge.com/summary.do?product=UA&doc=1&qid=70&SID=C4wLCyWhPfpFS8aSDPh&search_mode=GeneralSearch&update_back2search_link_param=yes) | (Course AND Quality AND Indicators AND Education) |
| # 43 | [258](http://apps.webofknowledge.com/summary.do?product=UA&doc=1&qid=71&SID=C4wLCyWhPfpFS8aSDPh&search_mode=GeneralSearch&update_back2search_link_param=yes) | Course AND Quality AND Indicators AND Education)  Refined by RESEARCH AREAS (EDUCATIONAL RESEARCH OR PSYCHOLOGY) |
| # 44 | [398](http://apps.webofknowledge.com/summary.do?product=UA&doc=1&qid=77&SID=C4wLCyWhPfpFS8aSDPh&search_mode=GeneralSearch&update_back2search_link_param=yes) | (Educational Program AND Quality AND Indicators AND Education)  Refined by RESEARCH DOMAINS (SOCIAL SCIENCES) AND   RESEARCH AREAS (EDUCATIONAL RESEARCH OR PSYCHOLOGY) AND DOCUMENT TYPES  ( ARTICLE ) |
| # 45 | [8](http://apps.webofknowledge.com/summary.do?product=UA&doc=1&qid=78&SID=C4wLCyWhPfpFS8aSDPh&search_mode=GeneralSearch&update_back2search_link_param=yes) | (Curriculum AND Excellence AND Indicators AND Education) |
| # 46 | [6](http://apps.webofknowledge.com/summary.do?product=UA&doc=1&qid=79&SID=C4wLCyWhPfpFS8aSDPh&search_mode=GeneralSearch&update_back2search_link_param=yes) | Curriculum AND Excellence AND Indicators AND Education)  Refined by RESEARCH DOMAINS (SOCIAL SCIENCES) AND RESEARCH AREAS (EDUCATIONAL RESEARCH) |
| # 47 | [1](http://apps.webofknowledge.com/summary.do?product=UA&doc=1&qid=80&SID=C4wLCyWhPfpFS8aSDPh&search_mode=GeneralSearch&update_back2search_link_param=yes) | (Syllabus AND Excellence AND Indicators AND Education) |
| # 48 | [17](http://apps.webofknowledge.com/summary.do?product=UA&doc=1&qid=83&SID=C4wLCyWhPfpFS8aSDPh&search_mode=GeneralSearch&update_back2search_link_param=yes) | (Educational Program AND Excellence AND Indicators AND Education) |
| # 49 | 0 | (Curriculum AND Excellence AND Markers AND Education) |
| # 50 | 0 | (Syllabus AND Excellence AND Markers AND Education) |
| # 51 | [151](http://apps.webofknowledge.com/summary.do?product=UA&doc=1&qid=86&SID=C4wLCyWhPfpFS8aSDPh&search_mode=GeneralSearch&update_back2search_link_param=yes) | (Curriculum AND Excellence AND Evaluation AND Education) |
| # 52 | 5 | (Syllabus AND Excellence AND Evaluation AND Education) |
| # 53 | [103](http://apps.webofknowledge.com/summary.do?product=UA&doc=1&qid=93&SID=C4wLCyWhPfpFS8aSDPh&search_mode=GeneralSearch&update_back2search_link_param=yes) | (Educational Program AND Excellence AND Evaluation AND Education)  Refined by AND RESEARCH AREAS: (EDUCATIONAL RESEARCH OR PSYCHOLOGY) AND DOCUMENT TYPES: (ARTICLE OR REVIEW) |
| # 54 | [2,151](http://apps.webofknowledge.com/summary.do?product=UA&doc=1&qid=100&SID=C4wLCyWhPfpFS8aSDPh&search_mode=GeneralSearch&update_back2search_link_param=yes) | (Curriculum AND Quality AND Evaluation AND Education)  Refined by:  RESEARCH AREAS: (EDUCATIONAL RESEARCH OR PSYCHOLOGY) AND DOCUMENT TYPES: (ARTICLE OR REVIEW) AND RESEARCH AREAS: (EDUCATIONAL RESEARCH OR PSYCHOLOGY) |
| # 55 | [58](http://apps.webofknowledge.com/summary.do?product=UA&doc=1&qid=101&SID=C4wLCyWhPfpFS8aSDPh&search_mode=GeneralSearch&update_back2search_link_param=yes) | (Syllabus AND Quality AND Evaluation AND Education |
| # 56 | [287](http://apps.webofknowledge.com/summary.do?product=UA&doc=1&qid=118&SID=C4wLCyWhPfpFS8aSDPh&search_mode=GeneralSearch&update_back2search_link_param=yes) | TOPIC: (indicators) *AND* TOPIC: (curriculum)  Refined by RESEARCH AREAS: (EDUCATIONAL RESEARCH OR PSYCHOLOGY) |
| # 57 | [152](http://apps.webofknowledge.com/summary.do?product=UA&doc=1&qid=123&SID=C4wLCyWhPfpFS8aSDPh&search_mode=GeneralSearch&update_back2search_link_param=yes) | TOPIC: (viability) AND TOPIC: (curriculum)  Refined by RESEARCH DOMAINS: (SOCIAL SCIENCES OR SCIENCE TECHNOLOGY) AND RESEARCH AREAS: (EDUCATIONAL RESEARCH OR HEALTH CARE SCIENCES SERVICES OR PSYCHOLOGY) AND RESEARCH AREAS: (EDUCATIONAL RESEARCH OR PSYCHOLOGY) |
