# Supplementary material for: Determining ‘curriculum viability’ through standards and inhibitors of curriculum quality: a scoping review
Source: BMC Med Educ. 2019 Sep 5;19:336. doi: 10.1186/s12909-019-1759-8 (PMC6727426; doi:10.1186/s12909-019-1759-8)

**Additional file 2: Assessing the quality of articles and Inter-rater agreement**

|  |  | **Questions (Qualsyst Criteria)** | | | | | | | | | | | | | | **Total score** | **%** | **Agreement** | **Quality** |
| --- | --- | --- | --- | --- | --- | --- | --- | --- | --- | --- | --- | --- | --- | --- | --- | --- | --- | --- | --- |
| **Study** | **Methodology** | **1** | **2** | **3** | **4** | **5** | **6** | **7** | **8** | **9** | **10** | **11** | **12** | **13** | **14** |  |  |  |  |
| **RK S1** | Quantitative Cross-sectional Survey | 2 | 1 | 2 | NA | NA | NA | NA | 2 | 2 | 1 | 0 | NA | 2 | 2 | 14/18 | 78 | ✓ | G |
| UM S1 |  | 2 | 1 | 2 | NA | NA | NA | NA | 1 | 2 | 1 | 0 | NA | 2 | 2 | 13/18 | 72 |  |  |
| **RK S2** | Systematic Review | 2 | 2 | 2 | NA | 2 | 2 | 2 | 1 | 2 | 0 |  |  |  |  | 15/18 | 83 | ✓ | H |
| UM S2 |  | 2 | 2 | 2 | NA | 2 | 2 | 2 | 1 | 2 | 0 |  |  |  |  | 15/18 | 83 |  |  |
| **RK S3** | Literature Review and Document Analysis | 2 | 1 | 2 | NA | NA | 1 | 1 | 0 | 2 | 0 |  |  |  |  | 9/16 | 56 | ✓ | S |
| UM S3 |  | 2 | 1 | 2 | NA | NA | 1 | 0 | 0 | 2 | 0 |  |  |  |  | 8/16 | 50 |  |  |
| **RK S4** | Qualitative exploratory study | 2 | 2 | 2 | NA | 2 | 2 | 2 | NA | 2 | 0 |  |  |  |  | 14/16 | 88 | x | G-H |
| UM S4 |  | 2 | 2 | 2 | NA | 2 | 2 | 2 | 0 | 2 | 0 |  |  |  |  | 14/18 | 78 |  |  |
| **RK S5** | Literature Review | 2 | 1 | 2 | 2 | NA | NA | 0 | NA | 2 | 1 |  |  |  |  | 10/16 | 63 | ✓ | S |
| UM S5 |  | 2 | 0 | 2 | 2 | NA | NA | 0 | NA | 2 | 1 |  |  |  |  | 9/16 | 56 |  |  |
| **RK S6** | Grounded theory and Delphi | 2 | 2 | 2 | 2 | 2 | 2 | 1 | 2 | 2 | NA |  |  |  |  | 16/18 | 89 | ✓ | H |
| UM S6 |  | 2 | 2 | 2 | 2 | 2 | 2 | 1 | 1 | 0 | NA |  |  |  |  | 15/18 | 83 |  |  |
| **RK S7** | Document Analysis and Framework Development | 2 | 1 | 2 | 1 | 1 | 2 | 2 | NA | 1 | 0 |  |  |  |  | 12/18 | 67 | ✓ | S |
| UM S7 |  | 2 | 1 | 2 | 1 | 0 | 2 | 1 | NA | 1 | 1 |  |  |  |  | 11/18 | 61 |  |  |
| **RK S8** | Evaluation Review | 2 | 0 | 2 | 1 | 1 | 1 | 2 | NA | 2 | 1 |  |  |  |  | 12/18 | 67 | ✓ | S |
| UM S8 |  | 2 | 0 | 2 | 1 | 1 | 1 | 2 | NA | 2 | 1 |  |  |  |  | 12/18 | 67 |  |  |
| **RK S9** | Systematic Review | 2 | 2 | 2 | 2 | 2 | 2 | 2 | 0 | 2 | 0 |  |  |  |  | 16/20 | 80 | ✓ | H |
| UM S9 |  | 2 | 2 | 2 | 1 | 2 | 2 | 2 | 1 | 2 | 1 |  |  |  |  | 17/20 | 85 |  |  |
| **RK S10** | Mixed Method  Qualitative-part | 2 | 2 | 2 | 0 | 2 | 2 | 2 | 1 | 1 | 0 |  |  |  |  | 14/20 | 70 | ✓ | S |
| UM S10 |  | 2 | 2 | 2 | 0 | 1 | 2 | 2 | 1 | 2 | 0 |  |  |  |  | 14/20 | 70 |  |  |
| RK 10 | Quantitative-part | 2 | 2 | 2 | 1 | NA | NA | NA | 1 | 2 | 2 | 1 | 0 | 2 | 2 | 17/22 | 77 | ✓ | G |
| UM 10 |  | 2 | 2 | 2 | 2 | NA | NA | NA | 1 | 2 | 2 | 0 | 0 | 2 | 2 | 17/22 | 77 |  |  |
| **RK S11** | Cross sectional Survey | 2 | 2 | 2 | 1 | NA | NA | 0 | 2 | 2 | 1 | 1 | 1 | 2 | 2 | 20/24 | 83 | ✓ | H |
| UM S11 |  | 2 | 2 | 2 | 2 | NA | NA | 0 | 2 | 1 | 2 | 2 | 0 | 2 | 2 | 20/24 | 83 |  |  |
| **RK S12** | Realist Review | 2 | 2 | 2 | 0 | 2 | 2 | 2 | 1 | 2 | 1 |  |  |  |  | 16/20 | 80 | x | G-H |
| UM S12 |  | 2 | 2 | 2 | 0 | 2 | 2 | 2 | 0 | 2 | 1 |  |  |  |  | 15/20 | 75 |  |  |
| **RK S13** | Qualitative Focus group | 2 | 2 | 2 | 0 | 2 | 2 | 2 | 0 | 2 | 0 |  |  |  |  | 14/20 | 70 | x | S |
| UM S13 |  | 2 | 2 | 2 | 0 | 2 | 2 | 2 | 1 | 2 | 0 |  |  |  |  | 15/20 | 75 |  |  |

(x = No agreement. ✓ = Agreement. Quality of article: H = High > 80%, Good = 71-79%, Sufficient = 50-70%, Limited = <50%.

**Qualsyst Criteria**

| **Quantitative Criteria** |
| --- |
| 1. Question / objective sufficiently described? |
| 1. Study design evident and appropriate? |
| 1. Method of subject/comparison group selection or source of information/input variables described and appropriate? |
| 1. Subject (and comparison group, if applicable) characteristics sufficiently described? |
| 1. If interventional and random allocation was possible, was it described? |
| 1. If interventional and blinding of investigators was possible, was it reported? |
| 1. If interventional and blinding of subjects was possible, was it reported? |
| 1. Outcome and (if applicable) exposure measure(s) well defined and robust to measurement / misclassification bias? means of assessment reported? |
| 1. Sample size appropriate? |
| 1. Analytic methods described/justified and appropriate? |
| 1. Some estimate of variance is reported for the main results? |
| 1. Controlled for confounding? |
| 1. Results reported in sufficient detail? |
| 1. Conclusions supported by the results? |

| **Qualitative Criteria** |
| --- |
| 1. Question / objective sufficiently described? |
| 1. Study design evident and appropriate? |
| 1. Context for the study clear? |
| 1. Connection to a theoretical framework / wider body of knowledge? |
| 1. Sampling strategy described, relevant and justified? |
| 1. Data collection methods clearly described and systematic? |
| 1. Data analysis clearly described and systematic? |
| 1. Use of verification procedure(s) to establish credibility? |
| 1. Conclusions supported by the results? |
| 1. Reflexivity of the account? |

Qualsyst Criteria

(0)

**Calculating Inter-rater Agreement (Cohen’s Kappa)**


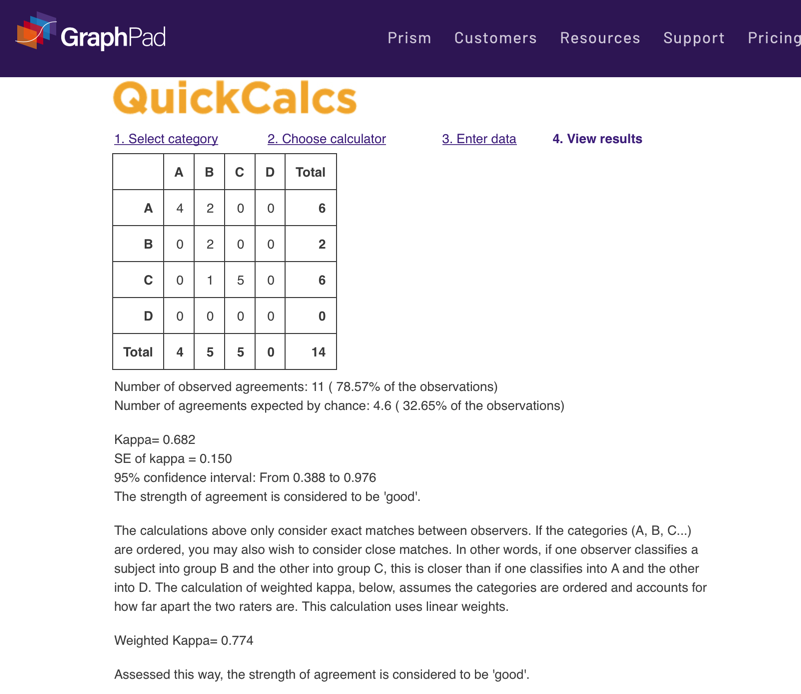

Supplement: Supplementary file 2 — Assessing the quality of articles and Inter-rater agreement. (DOCX 2207 kb) [file 12909_2019_1759_MOESM2_ESM.docx]
